# Recurrent *Granulibacter bethesdensis* Infections and Chronic Granulomatous Disease

**DOI:** 10.3201/eid1609.091800

**Published:** 2010-09

**Authors:** David E. Greenberg, Adam R. Shoffner, Adrian M. Zelazny, Michael E. Fenster, Kol A. Zarember, Frida Stock, Li Ding, Kimberly R. Marshall-Batty, Richard L. Wasserman, David F. Welch, Kishore Kanakabandi, Dan E. Sturdevant, Kimmo Virtaneva, Stephen F. Porcella, Patrick R. Murray, Harry L. Malech, Steven M. Holland

**Affiliations:** Author affiliations: National Institutes of Health, Bethesda, Maryland, USA (D.E. Greenberg, A.R. Shoffner, A.M. Zelazny, M.E. Fenster, K.A. Zarember, F. Stock, L. Ding, K.R. Marshall-Batty, P.R. Murray, H.L. Malech, S.M. Holland);; University of Texas Southwestern Medical Center, Dallas, Texas, USA (R.L. Wasserman, D.F. Welch);; Children's Medical Center, Dallas (D.F. Welch);; Medical City Dallas Hospital, Dallas (D.F. Welch);; National Institutes of Health, Hamilton, Montana, USA (K. Kanakabandi, D.E. Sturdevant, K. Virtaneva, S.F. Porcella)

**Keywords:** Bacteria, lymphadenitis, Granulibacter bethesdensis, Acetobacteraceae, relapse, chronic granulomatous disease, comparative genomic hybridization, CME, synopsis

## MEDSCAPE CME

Medscape, LLC is pleased to provide online continuing medical education (CME) for this journal article, allowing clinicians the opportunity to earn CME credit. This activity has been planned and implemented in accordance with the Essential Areas and policies of the Accreditation Council for Continuing Medical Education through the joint sponsorship of Medscape, LLC and Emerging Infectious Diseases. Medscape, LLC is accredited by the ACCME to provide continuing medical education for physicians. Medscape, LLC designates this educational activity for a maximum of 0.5 *AMA PRA Category 1 Credits*™. Physicians should only claim credit commensurate with the extent of their participation in the activity. All other clinicians completing this activity will be issued a certificate of participation. To participate in this journal CME activity: (1) review the learning objectives and author disclosures; (2) study the education content; (3) take the post-test and/or complete the evaluation at http://cme.medscape.com/viewpublication/30063; (4) view/print certificate.

## Learning Objectives

Upon completion of this activity, participants will be able to:

- Identify infectious organisms in cases of chronic granulomatous disease (CGD).
- Distinguish the clinical presentation of *Granulibacter bethesdensis* infection in CGD.
- Diagnose *G. bethesdensis* infection in CGD effectively.
- Plan effective treatment for *G. bethesdensis* infection in CGD.

## MEDSCAPE CME Editor

**Thomas J. Gryczan, MS, **Copyeditor, *Emerging Infectious Diseases. Disclosure: Thomas J. Gryczan, MS, has disclosed no relevant financial relationships.*

## MEDSCAPE CME AUTHOR

**Charles P. Vega, MD**, Associate Professor; Residency Director, Department of Family Medicine, University of California, Irvine. *Disclosure: Charles P. Vega, MD, has disclosed no relevant financial relationships.*

## AUTHORS

*Disclosures:* ***David E. Greenberg, MD; Adam R. Shoffner, BS; Adrian M. Zelazny, PhD; Michael E. Fenster, MD; Kol A. Zarember, PhD, BSc; Frida Stock, BS; Li Ding, MD; Kimberly R. Marshall-Batty, PhD, MS, BS; Kishore Kanakabandi; Dan E. Sturdevant, PhD; Kimmo Virtaneva; Stephen F. Porcella, PhD; Patrick R. Murray, PhD; Harry L. Malech, MD;*** *and* ***Steven M. Holland, MD,*** *have disclosed no relevant financial relationships.* ***Richard L. Wasserman, MD,*** *has disclosed the following relevant financial relationships: served as an advisor or consultant for Baxter Healthcare, CSL Behring, Talecris Biotherapeutics; served as a speaker or a member of a speakers bureau for Baxter Healthcare, CSL Behring, Talecris Biotherapeutics; received grants for clinical research from Baxter Healthcare, CSL Behring, Talecris Biotherapeutics, Nabi Biopharmaceuticals.* ***David F. Welch, PhD,*** *has disclosed the following relevant financial relationships: received grants for clinical research from Prodesse (Gen-Probe); owns stock, stock options, or bonds from Cepheid, Luminex Corporation, Merck & Co., Inc., Eli Lilly and Company, Boston Scientific Corporation, Becton, Dickinson and Company.*
