# Supplementary material for: Recurrent Granulibacter bethesdensis Infections and Chronic Granulomatous Disease
Source: Emerg Infect Dis. 2010 Sep;16(9):1341–8. doi: 10.3201/eid1609.091800 (PMC3294967; doi:10.3201/eid1609.091800)
Supplement: Related texts

# Review article: Recurrent *Granulibacter bethesdensis* Infections and Chronic Granulomatous Disease

**DOI:** 10.3201/eid1609.091800

**Authors:** David E. Greenberg, Adam R. Shoffner, Adrian M. Zelazny, Michael E. Fenster, Kol A. Zarember, Frida Stock, Li Ding, Kimberly R. Marshall-Batty, Richard L. Wasserman, David F. Welch, Kishore Kanakabandi, Dan E. Sturdevant, Kimmo Virtaneva, Stephen F. Porcella, Patrick R. Murray, Harry L. Malech, Steven M. Holland

**Affiliations:** Author affiliations: National Institutes of Health, Bethesda, Maryland, USA (D.E. Greenberg, A.R. Shoffner, A.M. Zelazny, M.E. Fenster, K.A. Zarember, F. Stock, L. Ding, K.R. Marshall-Batty, P.R. Murray, H.L. Malech, S.M. Holland);; University of Texas Southwestern Medical Center, Dallas, Texas, USA (R.L. Wasserman, D.F. Welch);; Children's Medical Center, Dallas (D.F. Welch);; Medical City Dallas Hospital, Dallas (D.F. Welch);; National Institutes of Health, Hamilton, Montana, USA (K. Kanakabandi, D.E. Sturdevant, K. Virtaneva, S.F. Porcella)

## Abstract

Chronic granulomatous disease (CGD) is characterized by frequent infections, most of which are curable. *Granulibacter bethesdensis* is an emerging pathogen in patients with CGD that causes fever and necrotizing lymphadenitis. However, unlike typical CGD organisms, this organism can cause relapse after clinical quiescence. To better define whether infections were newly acquired or recrudesced, we use comparative bacterial genomic hybridization to characterize 11 isolates obtained from 5 patients with CGD from North and Central America. Genomic typing showed that 3 patients had recurrent infection months to years after apparent clinical cure. Two patients were infected with the same strain as previously isolated, and 1 was infected with a genetically distinct strain. This organism is multidrug resistant, and therapy required surgery and combination antimicrobial drugs, including long-term ceftriaxone. *G. bethesdensis* causes necrotizing lymphadenitis in CGD, which may recur or relapse.

Chronic granulomatous disease (CGD) is a rare genetic disease caused by mutations in any of the 4 structural genes of the NADPH oxidase system and leads to defective production by phagocytes of superoxide and downstream oxygen metabolites (*1*). Infections in patients with CGD are caused by a narrow spectrum of pathogens, including *Staphylococcus aureus*, *Serratia marcescens*, *Burkholderia cepacia* complex, *Nocardia* spp., and *Aspergillus* spp (*2**–**4*). Although lymphadenitis is commonly encountered, a pathogen is isolated in only ≈60% of cases (*5*).

Most human bacterial infections, even those that are severe, are transient and curable. Bacteria such as *Mycobacterium tuberculosis* are unique human pathogens in part because of their ability to persist in a dormant state and reactivate later. The recurrent infections observed in patients with CGD, even when caused by the same species of organism, are the result of reinfection rather than relapse (*3**,**6*). *Granulibacter bethesdensis* is a recently described gram-negative bacterium in the family *Acetobacteraceae*; it has been isolated from 6 patients with CGD from North and Central America and Spain (*7**–**10*). The initial case was in an adult who had prolonged fever, necrotizing lymphadenitis, and multiple disease recurrences culminating in cure 2 years after seeking treatment. Persons with subsequent cases in the Americas had shorter periods before diagnosis and more rapid responses to therapy. A fatal case reported in Spain involved a patient with CGD in whom *G. bethesdensis* was the only pathogen identified. Given the increasing cases of this emerging pathogen, we present in greater detail the clinical course of these patients and molecular epidemiologic evidence to support the recurrent infections we have diagnosed for some of these patients.

Five patients were followed up at the National Institutes of Health (NIH) Clinical Center (Bethesda, MD, USA) under protocol 93-I-0119. Patients 2, 3, and 5 had been actively followed up at NIH for at least 8 years before receiving a diagnosis of *G. bethesdensis* infection. Patient 1 had been sent to NIH for evaluation of his lymphadenopathy and *Granulibacter* infection was diagnosed shortly thereafter. Patient 4 was referred to NIH for treatment and follow-up after his *Granulibacter* infection was diagnosed at an outside hospital (by R.L.W. and D.F.W.).

The *G. bethesdensis* high-density microarray platform, DNA microarray hybridization, and comparative genomic hybridization analysis used for typing of the *G. bethesdensis* isolates have been described (*9*). Bacterial DNA was isolated by using the NucliSens Kit (bioMérieux, Durham, NC, USA), and 16S rRNA genes from the 5 patient isolates were sequenced and analyzed as described (*8*). DNA was isolated from human tissue by using the Maxwell 16 Tissue DNA Purification Kit (Promega, Madison, WI, USA) according to the manufacturer’s protocol. DNA concentrations were measured by using a UV spectrophotometer (NanoDrop, Wilmington, DE, USA).

The 16S rRNA and methanol dehydrogenase subunit 1 (GeneID YP_744165.1) genes of *G. bethesdensis* were analyzed by using a PCR and primer sequences 16S-forward: 5′-TCGGGTGGGCACTCTAAAGG-3′, 16S-reverse: 5′-GCATCACTGCCTAGCTTCCC-3′, MDH-forward: 5′-CCGCAATACGGTCAATTCG-3′, and MDH-reverse: 5′-GCCGATCTTCCAGGTTTCTTC-3′. Each reaction mixture contained 47 μL of Platinum Blue PCR SuperMix (Invitrogen, Carlsbad, CA, USA) and 1.5 μL of each primer at a final concentration of 0.75 μmol/L, and the PCR was performed in a thermocycler (Eppendorf, Hauppauge, NY, USA). The PCR amplification conditions were 94°C for 10 min; 40 cycles at 94°C for 30 s, 60°C for 30 s, and 72°C for 30 s; and a final extension at 72°C for 5 min. PCR fragments were visualized by electrophoresis on a 2% agarose gel and showed the expected sizes of 137 bp (16SrRNA) and 63 bp (methanol dehydrogenase subunit 1).

## Case Reports

### Patient 1

A 39-year-old man from the United States with X-linked CGD had persistent fever, chills, weight loss, and increased levels of inflammatory markers starting in April 2003, as described (*5*). Computed tomography (CT) scans showed necrotic mediastinal and cervical nodes (Table A1). Resection samples of cervical nodes grew *G. bethesdensis*. Therapy with meropenem and doxycycline resulted in resolution of the lesions. However, the patient had recurrences of necrotizing cervical and axillary lymphadenitis over the next 2 years, and *G. bethesdensis* was isolated on 3 separate occasions. Treatment with ceftriaxone and doxycycline for 1 year resolved his lymphadenitis. He has had no further recurrence but has had persistent chronic fatigue since onset of infection.

### Patient 2

A 36-year-old man from the United States with X-linked CGD had cough and fever in November 2005. He had lymphadenopathy of the supraclavicular, paratracheal, subcarinal, perihilar, internal mammary, perigastric, retroperitoneal, iliac, and inguinal lymph nodes and multiple splenic lesions (Figure1, panel A; Table A1). The erythrocyte sedimentation rate (ESR) was 50 mm/h. Empirical treatment with itraconazole and linezolid did not prevent increased abdominal distension and continued fever.

**Figure 1:** Radiologic and histologic findings for patients with *Granulibacter bethesdensis* infections. A) Contrast-enhanced computed tomography (CT) image for patient 2, showing enlarged epigastric nodes (arrow) in the abdomen. B) Contrast-enhanced CT image for patient 3, showing lymphadenopathy (arrow). The heterogeneity of the lymph node suggests necrosis. C) Noncontrast CT image of the spine for patient 4, showing enlarged cervical lymph nodes (arrow). D) Contrast-enhanced CT image for patient 5, showing a left supraclavicular mass. Arrow highlights an area suggestive of necrosis. E) Hematoxylin and eosin–stained section from patient 4, showing necrotizing granulomatous inflammation in the excised node (original magnification ×10). F) Hematoxylin and eosin–­stained section from patient 2, showing necrotizing granulomas composed of epithelioid histiocytes, lymphocytes, and plasma cells from a perigastric node biopsy specimen (original magnification ×10).

A CT scan in December 2005 showed enlarging left internal mammary lymphadenopathy, ascites, and enlarging splenic lesions (Figure 2, panel A). Leukocyte count was 13.5 × 10^3^ cells/μL with 79% neutrophils. Ascitic fluid was cloudy with 3,100 leukocytes/μL (45% lymphocytes, 27% neutrophils) and 2,813 erythrocytes/μL. Gram stain showed no organisms. Treatment with meropenem and trimethoprim/sulfamethoxazole was begun. A CT-guided biopsy specimen of perigastric lymph nodes showed necrotizing granulomatous inflammation (Figure 1, panel F), but results of fungal and acid-fast staining were negative. Multiple blood cultures were negative. On hospital day 4, fever continued. Itraconazole was stopped, and voriconazole and tobramycin were started. Fever abated shortly thereafter and the patient recovered.

**Figure 2:** Radiologic and pathologic findings of *Granulibacter bethesdensis* infection in patient 2, a 36-year-old man from the United States with X-linked chronic granulomatous disease. A) Contrast-enhanced computed tomography (CT) image at initial examination (December 2005), showing multiple lucencies in the spleen (arrows) and edema and stranding in the omentum and mesentery. B) Contrast-enhanced CT image (September 2006), showing resolution of splenic lesions after prolonged antimicrobial drug therapy. C) Contrast-enhanced CT image (April 2008), showing multiple lucencies in the spleen consistent with abscesses. D) Gross view of the sectioned spleen after splenectomy (April 2008), showing numerous abscesses.

A gram-negative rod was isolated from ascitic fluid on Middlebrook 7H11 agar after 15 days of incubation; 16S rDNA sequencing confirmed that the isolate was *G. bethesdensis*. The patient was treated with ceftriaxone for 6 weeks and showed a positive response over the next year and resolution of his lesions (Figure 2, panel B). He continued to take trimethoprim/sulfamethoxazole and was treated with γ-interferon. In September 2007, sweats, chills, and splenic lesions developed. After CT-guided biopsy, he was treated with cefpodoxime and doxycycline; treatment with trimethoprim/sulfamethoxazole and γ-interferon continued.

One colony of a gram-negative rod was seen on Middlebrook 7H11 medium after 10 days of incubation; 16S rDNA sequencing confirmed that the isolate was *G. bethesdensis*. Despite antimicrobial drug therapy, the splenic lesions became more numerous and necessitated a splenectomy in April 2008 (Figure 2, panels C, D). A portion of splenic tissue was used for PCR detection of *Granulibacter*-specific 16S rRNA and methanol dehydrogenase genes (Figure 3). PCR products of the expected sizes were identified from splenic tissue. The culture from the splenectomy showed a positive result 6 days later on mycobacterial media. The patient was treated with tigecycline for 3 months and has had no further relapses.

**Figure 3:** Detection of *Granulibacter* sp. DNA by PCR of the spleen of a patient with chronic granulomatous disease. Lanes 1 and 2, no template controls for each primer set; lane 3, 400 ng of spleen DNA amplifying *Granulibacter* sp. 16S rRNA gene; lane 4, 400 ng of spleen DNA amplifying the *Granulibacter* sp. methanol dehydrogenase gene; lane 5, 100 ng DNA from the *G. bethesdensis* type strain amplifying the 16S rRNA gene (positive control); lane 6, 100 ng DNA from the *G. bethesdensis* type strain amplifying the methanol dehydrogenase gene (positive control). Left lane, molecular mass ladder. Expected PCR product sizes were 137 bp for the 16S rRNA gene and 63 bp for the methanol dehydrogenase gene. Values on the left are in bp.

### Patient 3

A 13-year-old boy from the United States with X-linked CGD was admitted for persistent fever and diffuse thoracic lymphadenopathy in January 2006. He had been followed up for *Staphylococcus epidermidis* submandibular abscesses since November 2004. He had enlarged nodes, many of which appeared necrotic, in the superior mediastinum, pretracheal region, aortopulmonary window, right hilum and subcarinal areas (Figure 1, panel B; Table A1). A transbronchial biopsy specimen of the subcarinal and right paratracheal nodes grew 1 colony each of *Candida glabrata*, *S. epidermidis*, *Streptococcus mitis* group, and a gram-negative rod. The gram-negative rod grew after 4 days of incubation on buffered-charcoal yeast extract (BCYE) agar. Routine biochemical tests did not identify the organism, but 16S rDNA sequencing confirmed that the isolate was *G. bethesdensis*.

The patient was initially treated with meropenem and voriconazole. When a repeat CT scan 2 weeks later showed enlarged mediastinal lymph nodes, meropenem was discontinued and ceftriaxone, tobramycin, and doxycycline were initiated. A CT scan 1 week later showed smaller lymph nodes. The patient stopped taking tobramycin after renal toxicity developed in March 2006, and he received ceftriaxone until May 2006. His lymphadenopathy resolved and he received doxycycline until March 2007. In December 2008, new fevers and subcarinal lymphadenopathy developed. A fine-needle aspiration specimen yielded a gram-negative rod on fungal media (IMA agar) after 15 days of incubation; 16S rDNA sequencing confirmed that the isolate was *G. bethesdensis*. He responded clinically and radiographically to ceftriaxone. He was then switched to cefdinir in January 2009 after gallstones developed and continued to receive this therapy until July 2009. The patient has had no further relapses.

### Patient 4

A 17-year-old boy from Panama with X-linked CGD had cervical lymphadenopathy for 2 months in June 2005. He had been treated with trimethoprim/sulfamethoxazole until the age of 15 when a chest wall infection with an unidentified basidiomycete developed. Since then, he had responded well to itraconazole and trimethoprim/sulfamethoxazole prophylaxis until lymphadenopathy developed. Cervical and mediastinal lymphadenopathy biopsy specimens (Figure 1, panel C) showed necrotizing granulomatous inflammation. One week after surgery, painful hard, 1-cm, right anterior cervical and 2-cm, right supraclavicular lymph nodes developed. Treatment with levofloxacin was begun, and another lymph node was excised and cultured. Necrotizing granulomata were seen again (Figure 1, panel E).

Pinpoint colonies were seen on sheep blood agar after 6 days of incubation and were subcultured in BCYE medium. The gram-negative rod could not be identified biochemically; 16S rDNA sequencing confirmed that the isolate was *G. bethesdensis*. While receiving levofloxacin, adenopathy increased in the patient. Five weeks later, another cervical lymph node was removed. After 4 days, the culture grew *G. bethesdensis* as pinpoint colonies on sheep blood agar. The patient was treated with doxycycline and responded well clinically without further recurrences.

### Patient 5

The 37-year-old brother of patient 2, who also had X-linked CGD, had fever, chills, and chest pain for 2 weeks in October 2008. New left lower lobe nodules and left infrahilar adenopathy prompted bronchoalveolar lavage, which showed macrophages and scattered neutrophils. Cultures were negative, but the patient was treated with levofloxacin, linezolid and posaconazole and continued receiving trimethoprim/sulfamethoxazole. Fever and pulmonary nodules resolved but adenopathy persisted. Treatment with antimicrobial drugs was stopped in December 2008. Fever and chills returned in February 2009. His ESR was 78 mm/h. Magnetic resonance imaging of the chest showed multiple enlarged anterior mediastinal and bilateral hilar lymph nodes. Bronchoscopy with transbronchial needle aspiration yielded *S. epidermidis* and α-hemolytic streptococci in liquid media. The patient was then treated with voriconazole and ceftriaxone.

Despite this therapy, left supraclavicular lymphadenopathy and splenic and liver lesions developed in April 2009 (Figure 1, panel D; Table A1). A lymph node biopsy specimen in May 2009 showed necrotizing granulomatous inflammation and grew *G. bethesdensis* after 7 days of incubation on BCYE and mycobacterial media. Ototoxicity developed while he was being treated with ceftriaxone, gentamicin, and vancomycin. He was then treated with ceftriaxone, doxycycline, and trimethoprim/sulfamethoxazole for 4 months and showed clinical and radiographic improvement. He was treated with cefdinir in September 2009 and has continued treatment with doxycycline and trimethoprim/sulfamethoxazole and has not had any new relapses. Patients 2 and 5 live apart but see each other a few times per year and had been together during the illness of patient 2.

All patients had fever, lymphadenopathy, and increased ESRs for weeks or months (Table A1). The chronicity of the prodrome is distinct from the more typical clinical appearance of staphylococcal lymphadenitis in CGD, which is frequently cervical and of shorter duration before being observed. All biopsy tissues showed necrotizing granulomatous inflammation but staining did not detect any organisms. However, organisms are not usually abundant in persons with CGD lymphadenitis and stains are often not diagnostic. All samples grew *G. bethesdensis* within 3 weeks on a variety of media (Middlebrook 7H11, BCYE, and fungal media), but growth was sparse. PCR amplification identified specific *G. bethesdensis* bands in excised spleen, which indicated that molecular diagnosis can be made from fresh tissue, a useful consideration for an organism that is slow growing and difficult to identify (Figure 3).

Those patients who received ceftriaxone had good clinical responses. However, patients appeared to have responded to other agents, including meropenem, aminoglycosides, doxycycline, and trimethoprim/sulfamethoxazole in various combinations. Patients 1 and 2 had recurrences over prolonged periods, which indicated a capacity for *G. bethesdensis* to survive in a clinically latent state.

The 16S rDNA sequences for all patients showed 100% matches to the 16S rDNA sequence derived from the isolate from patient 1, NIHCGD1, the type strain of *G. bethesdensis* (GenBank accession no. AY788950). This finding indicated that these isolates are all appropriately designated *G. bethesdensis* sensu stricto. The isolate reported by Lopez et al. (*10*) from Spain showed only a 99.7% match for the 16S rDNA sequence, which suggested regional variability or another *Granulibacter* sp.

We had shown that all isolates from patient 1 over a 2-year period were identical (*9*). DNA samples from 3 isolates from patient 2 obtained over a 3-year period were hybridized to the previously reported *Granulibacter* microarray chip (*9*). Hybridization patterns of the 3 isolates were essentially identical, which indicated that the same organism was responsible for disease in patient 2 from his initial hospitalization through his splenectomy (Figure 4). This comprehensive analysis of genomic stability over several years indicates that *G. bethesdensis* can potentially achieve clinical latency over prolonged periods in the human host, even without causing signs or symptoms.

**Figure 4:** Physical gene representation of DNA hybridization of 11 isolates of *Granulibacter bethesdensis* by DNA–DNA hybridization microarray-based investigation of gene distributions among *G. bethesdensis* isolates. Every open reading frame in the *G. bethesdensis* type strain is represented. Hybridization is shown in yellow, and absence of hybridization is shown in blue. Four sequential isolates (NIH 1.1, NIH 1.2, NIH 1.3, and NIH 1.4) from patient 1 are shown from left to right and group with an identical hybridization pattern. Three sequential isolates (NIH 2.1, NIH 2.2, and NIH 2.3) from patient 2 are shown from left to right and demonstrate virtually identical patterns of hybridization. Two isolates (NIH 3.1 and NIH 3.2) from patient 3 have different hybridization patterns. One isolate (NIH 4.1) from patient 4 has a unique hybridization pattern. One isolate (NIH 5.1) from patient 5 is distinct from that of his brother (patient 2). Dates that each infected tissue sample were obtained are listed. NIH, National Institutes of Health.

In contrast to DNA from patient 2, DNA from the second isolate from patient 3 was compared with that from his original isolate by using the *Granulibacter* microarray chip. The hybridization pattern of his second isolate was distinct from his first isolate at multiple loci of the genome, which indicated reinfection with a different strain of *G. bethesdensis* (Figure 4). The *G. bethesdensis* isolate from patient 5 was distinct from that of his brother (patient 2) at multiple loci, as determined by using the *Granulibacter* microarray chip (Figure 4).

## Conclusions

Five patients with CGD from different parts of North and Central America had strikingly similar syndromes of prolonged fever and necrotizing lymphadenitis, all of which were caused by *G. bethesdensis.* Unlike most bacteria, *G. bethesdensis* can cause acute infections, relapses after apparently effective therapy, and reinfections, which indicate a lack of sterilizing and protective immunity in persons with CGD. These features are uncommon but serious. Tuberculosis, for example, causes acute disease, relapsing disease after prolonged latency and reinfection after definitive treatment. Sterilizing immunity has been difficult to prove (*11**,**12*). In contrast to most other CGD pathogens, most disseminated *G. bethesdensis* infections were chronic but not fatal. Although none of the 5 patients reported here died from their *G. bethesdensis* infections, a child from Spain died from infection with a *G. bethesdensis* isolate that was slightly different in its 16S rDNA sequence from the reference strain (*10*). Similar to our experience with humans, infection with *G. bethesdensis* in mice with CGD leads to a chronic persistent infection with a paucity of symptoms (*7*).

The *Granulibacter* microarray has enabled us to definitively identify genetic variability in *G. bethesdensis* through comparative genomic hybridization. Although all patient isolates were phenotypically *Granulibacter* spp. and 100% identical on the basis of full-length 16S rDNA sequencing, most of the hybridization patterns of patient isolates were different and unique according to comparative genomic hybridization, which indicated that these isolates are potentially distinct strains (*9*). This capacity proved critical for identifying persistence (the 4 isolates from patient 1 and the 3 isolates of patient 2) and reinfection (patient 3). Therefore, for patients 1 and 2, *Granulibacter* spp. most likely resided within the host in clinically latent reservoirs.

The ability to recur months to years after apparent clinical improvement is unlike other bacterial pathogens in persons with CGD. Use of comparative genomic hybridization to type our strains was critical in differentiating relapse from reinfection and in reinforcing our approach to treat these persons with these infections with long courses of antimicrobial drugs. Patients 1 and 2 may have had recurring environmental exposure to a source of nearly genetically identical *G. bethesdensis*, but this suggestion cannot be proven because we have not identified the environmental source for any of the isolates obtained. Patient 3 may have had a mixed infection at his initial hospitalization, followed by clearance of 1 isolate of *Granulibacter* spp. and persistence of the other. The microarray comparative genomic hybridization data demonstrate that for patients 2 and 5, brothers with splenic disease, each had different isolates.

Isolation of *G. bethesdensis* is time-consuming, growth is consistently sparse, and initial definitive identification requires sequencing the 16S rRNA gene. In the clinical laboratory, *G. bethesdensis* was most frequently isolated on special media and not on routine media normally used for cultivating gram-negative bacteria. Therefore, development of a molecular test for *G. bethesdensis* is imperative and will improve the speed and sensitivity of diagnosis. PCR of fresh tissue with 2 highly specific gene probes accurately detected *Granulibacter* spp. DNA in the spleen of patient 2. Development of PCR technology for fixed specimens is ongoing.

Members of the family *Acetobacteraceae* have been isolated from various tropical fruits, fermented foods, flowers, and soil (*13*). Although *G. bethesdensis* has thus far been isolated only from patients with CGD, other members of the family *Acetobacteraceae* appear to be emerging as pathogens in non-CGD hosts. Within the past 5 years, infections with other *Acetobacteraceae* spp. in 6 patients have been described. *Acetobacter cibinongensis* was found in the blood of a patient with end-stage renal disease and fever (*14*). *Acetobacter indonesiensis* was found in the sputum of a lung transplant patient (*15*). *Asaia bogorensis* has been reported as a cause of bacteremia in 2 intravenous drug users (*16**,**17*) and as a cause of peritonitis in a patient receiving peritoneal dialysis (*18*). *Asaia lannaensis* was described as a cause of bacteremia in a bone marrow transplant patient (*19*). It is unclear whether the increasing recognition of these opportunistic pathogens has been caused by improved microbiologic detection or by a yet unrecognized shift in our interactions with the microbial world. Why *G. bethesdensis* appears to cause clinical disease in the CGD host specifically is the subject of ongoing laboratory studies.

*G. bethesdensis* is resistant to antimicrobial drugs in vitro and apparently in vivo. Susceptibility testing is difficult because of the inherent slow growth of this pathogen and the lack of established susceptibility criteria. This organism shows extensive resistance to most cephalosporins, penicillins (including carbapenems), and quinolones. However, ceftriaxone (MIC 8 μg/mL), aminoglycosides (e.g., gentamicin, MIC 8 μg/mL), doxycycline, and trimethoprim/sulfamethoxazole show activity in vitro. Our patients were treated with various combinations of these drugs; all showed initial resolution of disease. Ceftriaxone is currently our preferred treatment.

The reasons underlying the emergence of new pathogens are complex. The changing environments in which we and our microbial companions live make predicting when, where, and why a new pathogen will emerge difficult. Typically, we are best at recognizing clinical syndromes and working back toward the etiology. Given the difficulty of growing and identifying *Granulibacter* spp., other cases of this infection in patients with CGD and those without CGD may have been overlooked. To address this issue, we now concentrate specimens, particularly from lymph nodes of patients with CGD, by centrifugation, place the specimens on BCYE and Middlebrook 7H11 agar, and incubate cultures for a minimum of 2 weeks.

Clinical management of CGD has long been confounded by the relatively high rate of failure of isolating a pathogen from involved sites. This failure has been attributed to many features of CGD, including exuberant inflammation. In some cases, clinicians attribute culture-negative lesions to the inflammatory diathesis of CGD. However, the finding of a difficult-to-grow and difficult-to-identify pathogen makes this assumption unlikely, particularly in the setting of necrotizing lymphadenopathy. Identification of a fastidious, slow-growing bacterium that causes a discrete, chronic infection reminds us that new syndromes remain to be identified, even within well-described and well-characterized diseases.

## Appendix

**Table A1:** Features of 5 patients with *Granulibacter bethesdensis* infection*

Patient no.	Age, y/sex	Country of origin	Radiographic findings	Pathologic findings (site)	Leukocytes, × 10^3^ cells/μL†	ESR, mm/h†	Duration of signs and symptoms, wk‡	Treatment§	Recurrence
1	39/M	USA	Cervical, mediastinal lymphadenopathy; splenic lesions	Necrotizing granulomatous inflammation (cervical lymph nodes)	11.3	90	20	**Meropenem**; **doxycycline**; TMP/SMX; **ceftriaxone**	Relapse
2	36/M	USA	Cervical, mediastinal, abdominal lymphadenopathy	Necrotizing granulomatous inflammation (perigastric lymph node)	10.2	50	8	TMP-SMX; **meropenem; tobramycin; ceftriaxone**; doxycycline; cefpodoxime; γ-interferon; tigecycline	Relapse
3	13/M	USA	Cervical, mediastinal, lymphadenopathy	Lymphohistiocytic infiltrate (transtracheal lymph node biopsy specimen)	5.9	44	4	**Ceftriaxone**; **tobramycin**; **doxycycline**; cefdinir	Reinfection
4	17/M	Panama	Cervical, mediastinal lymphadenopathy	Necrotizing granulomatous inflammation (cervical lymph node)	NA	NA	8	Doxycycline	No
5	37/M	USA	Cervical, mediastinal lymphadenopathy; liver and splenic lesions	Necrotizing granulomatous inflammation (supraclavicular lymph node)	7.3	55	16	**Ceftriaxone**; gentamicin; doxycycline; TMP/SMX; cefdinir	No

*All patients have X-linked chronic granulomatous disease. ESR, erythrocyte sedimentation rate; TMP/SMX, trimethoprim/sulfamethoxazole; NA, not available. †At time of patient visit or diagnosis of *Granulibacter* spp. infection at the National Institutes of Health, Bethesda, Maryland, USA. ‡From onset of symptoms to culture-confirmed infection; initial symptoms in those with recurrence. §Drugs used specifically for treatment of *G. bethesdensis* infection alone or in combination during the patient’s illness. Drugs in **boldface** were those for which the largest clinical response was observed in that patient.

## References

[1] Segal BH, Leto TL, Gallin JI, Malech HL, Holland SM. Genetic, biochemical, and clinical features of chronic granulomatous disease. Medicine (Baltimore). 2000;79:170–200. 10.1097/00005792-200005000-0000410844936

[2] Dorman SE, Guide SV, Conville PS, DeCarlo ES, Malech HL, Gallin JI, *Nocardia* infection in chronic granulomatous disease. Clin Infect Dis. 2002;35:390–4. 10.1086/34141612145721

[3] Guide SV, Stock F, Gill VJ, Anderson VL, Malech HL, Gallin JI, Reinfection, rather than persistent infection, in patients with chronic granulomatous disease. J Infect Dis. 2003;187:845–53. 10.1086/36838812599059

[4] Speert DP, Bond M, Woodman RC, Curnutte JT. Infection with *Pseudomonas cepacia* in chronic granulomatous disease: role of nonoxidative killing by neutrophils in host defense. J Infect Dis. 1994;170:1524–31. 10.1093/infdis/170.6.15247527826

[5] Winkelstein JA, Marino MC, Johnston RB Jr, Boyle J, Curnutte J, Gallin JI, Chronic granulomatous disease. Report on a national registry of 368 patients. Medicine (Baltimore). 2000;79:155–69. 10.1097/00005792-200005000-0000310844935

[6] Greenberg DE, Goldberg JB, Stock F, Murray PR, Holland SM, Lipuma JJ. Recurrent *Burkholderia* infection in patients with chronic granulomatous disease: 11-year experience at a large referral center. Clin Infect Dis. 2009;48:1577–9. 10.1086/59893719400745PMC2850592

[7] Greenberg DE, Ding L, Zelazny AM, Stock F, Wong A, Anderson VL, A novel bacterium associated with lymphadenitis in a patient with chronic granulomatous disease. PLoS Pathog. 2006;2:e28. 10.1371/journal.ppat.002002816617373PMC1435791

[8] Greenberg DE, Porcella SF, Stock F, Wong A, Conville PS, Murray PR, *Granulibacter bethesdensis* gen. nov., sp. nov., a distinctive pathogenic acetic acid bacterium in the family *Acetobacteraceae.* Int J Syst Evol Microbiol. 2006;56:2609–16. 10.1099/ijs.0.64412-017082400

[9] Greenberg DE, Porcella SF, Zelazny AM, Virtaneva K, Sturdevant DE, Kupko JJ III, Genome sequence analysis of the emerging human pathogenic acetic acid bacterium *Granulibacter bethesdensis.* J Bacteriol. 2007;189:8727–36. 10.1128/JB.00793-0717827295PMC2168926

[10] Lopez FC, de Luna FF, Delgado MC, de la Rosa II, Valdezate S, Nieto JA, *Granulibacter bethesdensis* isolated in a child patient with chronic granulomatous disease. J Infect. 2008;57:275–7. 10.1016/j.jinf.2008.04.01118558432

[11] Andrews JR, Gandhi NR, Moodley P, Shah NS, Bohlken L, Moll AP, Exogenous reinfection as a cause of multidrug-resistant and extensively drug-resistant tuberculosis in rural South Africa. J Infect Dis. 2008;198:1582–9. 10.1086/59299118847372

[12] Jasmer RM, Bozeman L, Schwartzman K, Cave MD, Saukkonen JJ, Metchock B, Recurrent tuberculosis in the United States and Canada: relapse or reinfection? Am J Respir Crit Care Med. 2004;170:1360–6. 10.1164/rccm.200408-1081OC15477492

[13] Seearunruangchai A, Tanasupawat S, Keeratipibul S, Thawai C, Itoh T, Yamada Y. Identification of acetic acid bacteria isolated from fruits collected in Thailand. J Gen Appl Microbiol. 2004;50:47–53. 10.2323/jgam.50.4715057711

[14] Gouby A, Teyssier C, Vecina F, Marchandin H, Granolleras C, Zorgniotti I, *Acetobacter cibinongensis* bacteremia in human. Emerg Infect Dis. 2007;13:784–5.1804404210.3201/eid1305.060532PMC2738439

[15] Bittar F, Reynaud-Gaubert M, Thomas P, Boniface S, Raoult D, Rolain JM. *Acetobacter indonesiensis* pneumonia after lung transplant. Emerg Infect Dis. 2008;14:997–8. 10.3201/eid1406.07123618507930PMC2600273

[16] Tuuminen T, Roggenkamp A, Vuopio-Varkila J. Comparison of two bacteremic *Asaia bogorensis* isolates from Europe. Eur J Clin Microbiol Infect Dis. 2007;26:523–4. 10.1007/s10096-007-0313-517562089

[17] Tuuminen T, Heinasmaki T, Kerttula T. First report of bacteremia by *Asaia bogorensis*, in a patient with a history of intravenous-drug abuse. J Clin Microbiol. 2006;44:3048–50. 10.1128/JCM.00521-0616891542PMC1594642

[18] Snyder RW, Ruhe J, Kobrin S, Wasserstein A, Doline C, Nachamkin I, *Asaia bogorensis* peritonitis identified by 16S ribosomal RNA sequence analysis in a patient receiving peritoneal dialysis. Am J Kidney Dis. 2004;44:e15–7. 10.1053/j.ajkd.2004.04.04215264206

[19] Abdel-Haq N, Savasan S, Davis M, Asmar BI, Painter T, Salimnia H. *Asaia lannaensis* bloodstream infection in a child with cancer and bone marrow transplantation. J Med Microbiol. 2009;58:974–6. 10.1099/jmm.0.008722-019502367

# Review article: Recurrent *Granulibacter bethesdensis* Infections and Chronic Granulomatous Disease

**DOI:** 10.3201/eid1609.091800

**Authors:** David E. Greenberg, Adam R. Shoffner, Adrian M. Zelazny, Michael E. Fenster, Kol A. Zarember, Frida Stock, Li Ding, Kimberly R. Marshall-Batty, Richard L. Wasserman, David F. Welch, Kishore Kanakabandi, Dan E. Sturdevant, Kimmo Virtaneva, Stephen F. Porcella, Patrick R. Murray, Harry L. Malech, Steven M. Holland

**Affiliations:** Author affiliations: National Institutes of Health, Bethesda, Maryland, USA (D.E. Greenberg, A.R. Shoffner, A.M. Zelazny, M.E. Fenster, K.A. Zarember, F. Stock, L. Ding, K.R. Marshall-Batty, P.R. Murray, H.L. Malech, S.M. Holland);; University of Texas Southwestern Medical Center, Dallas, Texas, USA (R.L. Wasserman, D.F. Welch);; Children's Medical Center, Dallas (D.F. Welch);; Medical City Dallas Hospital, Dallas (D.F. Welch);; National Institutes of Health, Hamilton, Montana, USA (K. Kanakabandi, D.E. Sturdevant, K. Virtaneva, S.F. Porcella)

## Earning CME Credit

To obtain credit, you should first read the journal article. After reading the article, you should be able to answer the following, related, multiple-choice questions. To complete the questions and earn continuing medical education (CME) credit, please go to http://cme.medscape.com/viewpublication/30063. Credit cannot be obtained for tests completed on paper, although you may use the worksheet below to keep a record of your answers. You must be a registered user on Medscape.com. If you are not registered on Medscape.com, please click on the New Users: Free Registration link on the left hand side of the website to register. Only one answer is correct for each question. Once you successfully answer all post-test questions you will be able to view and/or print your certificate. For questions regarding the content of this activity, contact the accredited provider, CME@medscape.net. For technical assistance, contact CME@webmd.net. American Medical Association’s Physician’s Recognition Award (AMA PRA) credits are accepted in the US as evidence of participation in CME activities. For further information on this award, please refer to http://www.ama-assn.org/ama/pub/category/2922.html. The AMA has determined that physicians not licensed in the US who participate in this CME activity are eligible for *AMA PRA Category 1 Credits*™. Through agreements that the AMA has made with agencies in some countries, AMA PRA credit is acceptable as evidence of participation in CME activities. If you are not licensed in the US and want to obtain an AMA PRA CME credit, please complete the questions online, print the certificate and present it to your national medical association.

## Article Title: Recurrent *Granulibacter bethesdensis* Infections and Chronic Granulomatous Disease

## CME Questions

**1. A 35-year-old man presents with a history of X-linked chronic granulomatous disease (CGD) in the hospital. He was admitted after 3 weeks of fever. Physical examination reveals obvious lymphadenopathy. Laboratory evaluation reveals an elevated erythrocyte sedimentation rate, but a Gram stain for organisms on 1 of his lymph nodes is negative.This patient is most likely infected with which of the following organisms?**A. Group B *Streptococcus *speciesB. *Mycoplasma pneumoniae*C. *Aspergillus *speciesD. *Campylobacter jejuni***2. Which of the following statements about a possible infection with *Granulibacter bethesdensis *in this patient is most accurate?**A. This infection is unlikely, given the patient’s duration of feverB. Lymphadenopathy was present in all cases of infection with *G. bethesdensis*C. Infection with *G. bethesdensis *does not promote an elevation in the erythrocyte sedimentation rateD. *G. bethesdensis *was evident on bacterial staining in all cases**3. The patient has a battery of tests and procedures to establish a causative organism for his infection. Which of the following statements about the diagnosis of *G. bethesdensis *infection is most accurate?**A. *G. bethesdensis *cultures grew readily, but only on Middlebrook mediaB. PCR amplification testing of excised tissue could identify *G. bethesdensis*C. All infections with *G. bethesdensis *could be traced to the same strainD. There was no evidence of reactivation of latent infection**4. A bacterial culture from your patient is now growing *G. bethesdensis. *Which of the following statements about his treatment and prognosis is most accurate?**A. Ceftriaxone is a good choice of treatmentB. Macrolide antibiotics are particularly effective for treating *G. bethesdensis*C. Disseminated *G. bethesdensis* infection was uniformly fatalD. *G. bethesdensis* responds to only 1 or 2 antibiotics

## Activity Evaluation

**Table:** 

1. The activity supported the learning objectives.				
Strongly Disagree				Strongly Agree
1	2	3	4	5
2. The material was organized clearly for learning to occur.				
Strongly Disagree				Strongly Agree
1	2	3	4	5
3. The content learned from this activity will impact my practice.				
Strongly Disagree				Strongly Agree
1	2	3	4	5
4. The activity was presented objectively and free of commercial bias.				
Strongly Disagree				Strongly Agree
1	2	3	4	5
